# Ocular Dominance Plasticity of Areas 17 and 21a in the Cat

**DOI:** 10.3389/fnins.2019.01039

**Published:** 2019-10-11

**Authors:** Jian Wang, Zheyi Ni, Anqi Jin, Tiandong Yu, Hongbo Yu

**Affiliations:** School of Life Sciences, State Key Laboratory of Medical Neurobiology, Collaborative Innovation Center for Brain Science, Fudan University, Shanghai, China

**Keywords:** ocular dominance plasticity, critical period, hierarchical neural network, area 21a, optical imaging

## Abstract

The visual system is organized in a parallel and hierarchical architecture. However, the plasticity in hierarchical neural networks is controversial across different response features and at different levels. In this study, we recorded areas 17 and 21a, earlier and intermediate stages of the visual cortex in the cat, respectively, by single-unit recording and intrinsic-signal optical imaging. We found that ocular dominance (OD) plasticity evoked by monocular deprivation (MD) was stronger in area 21a than in area 17 in the critical period (CP), and this plasticity became weaker but still persisted in area 21a while it disappeared in area 17 beyond the CP. These results suggest a diversified functional plasticity along the visual information processing pathways in a hierarchical neural network.

## Introduction

Hierarchical architecture is a hallmark of the organization of the visual system. Information from the retina is relayed by the lateral geniculate nucleus (LGN) and conveyed to the primary visual cortex and extrastriate visual areas in serial order. Neurons integrate across greater spatial and temporal extents along the hierarchy (Chaudhuri et al., 2015), and sophisticated properties emerge at higher levels through the build-up of simpler properties at lower levels (Nassi and Callaway, 2009). The properties of plasticity are distinctive in hierarchical neural networks. The pioneering works of Hubel and Wiesel have already demonstrated this at lower hierarchical levels, where they showed that ocular dominance (OD) plasticity spans only a few weeks in the primary visual cortex of the kittens. This plasticity is most robust during a special time window called the critical period (CP) and diminishes as the cat grows older (Hubel and Wiesel, 1970).

On the other hand, it is also known that high-level ventral streams are capable of modifying their connectivity throughout life to address experience-dependent changes in perception (Gilbert and Li, 2012). The inferior temporal (IT) cortex in the macaque monkey is characterized by its early-emerging adult-like patterns of responsiveness and protracted developmental phase (Rodman, 1994). Individual IT neurons could distinguish behaviorally relevant stimuli after brief periods of training (Jagadeesh et al., 2001). Plasticity for acquiring the face/non-face categorization ability was even preserved in early-blind individuals who gained sight late in childhood after treatment for dense bilateral cataracts (Gandhi et al., 2017).

The notion of the CP in the primary visual cortex and the evidence of life-long functional plasticity in the high-level visual cortex seem to be controversial, but this might only be the reflection of diversified plasticity in a hierarchical system. For example, step-by-step enhancement (cascading effect) of the visual adaptation effect was proposed from a line of studies, which suggests that plasticity accumulates along the bottom–up stream (Kohn and Movshon, 2003; Dhruv and Carandini, 2014). This idea is in line with some previous monocular deprivation (MD) studies. Within the cortical microcircuits of the primary visual cortex, the OD of neurons in layer IV had a CP ending earlier than the CP for layers II, III, V, and VI after MD (Daw et al., 1992), and 24 h’ MD could evoke strong OD shifts at other layers except for layer IV (Trachtenberg et al., 2000), since visual signals are thought to propagate primarily from layer IV (bottom) to other layers (up). In contrast, some other investigations are not consistent with this cascading idea. As to the hierarchy across different cortical regions, the orientation adaptation effect was not enhanced in the posterior medial lateral suprasylvian (PMLS) area compared to the lower-level area 17 (Li et al., 2017). Regarding OD plasticity, the CP was found to be over sooner in the lateral suprasylvian (LS) visual area, an extrastriate visual area in the cat specialized for motion processing, than in area 17 (Jones et al., 1984). Since PMLS and LS are thought to be in the dorsal stream of the visual system (Payne, 1993; Dreher et al., 1996), it is arguable that these conflicting results might not apply to the ventral pathway. In fact, in strabismic cats, area 21a, as the gateway of the ventral pathway, is more affected in amblyopia than the PMLS area, while area 18 seems to remain unaffected (Schroder et al., 2002).

Here, we chose areas 17 and 21a in cats, which belong to the early and intermediate ventral streams of the visual pathway, respectively, to investigate the development of OD plasticity in the hierarchical system. Area 21a is located in the middle part of the caudal suprasylvian gyrus, recurrently connecting with areas 17, 18 and 19 (Michalski et al., 1993; Morley et al., 1997). It is presumed to be a homolog of primate V4, which is the gateway of the ventral pathway in primates (Dreher et al., 1993, 1996; Payne, 1993). The neurons in area 21a have unique eye preferences, with an overall broad distribution from monocularly to binocularly driven responses (Dreher et al., 1993). Similar to area 17, neurons in area 21a with the same preferred orientations are functionally organized into a slab-like columnar structure (Huang et al., 2006; Villeneuve et al., 2009), making it practical to use the same stimuli that have been used to examine plasticity in area 17.

Nevertheless, to date, the details of the OD plasticity in area 21a is unknown. In this study, intrinsic signal optical imaging and single-unit recordings were applied to examine hemodynamic signals and action potentials in areas 17 and 21a. We quantitatively analyzed the OD shift of area 21a during and beyond the CP and compared the results to those obtained from area 17. Here, we reported that OD plasticity was stronger in area 21a than in area 17 in the CP and persisted in area 21a but disappeared in area 17 beyond the CP.

## Materials and Methods

### Animal Preparation

Nineteen kittens (5 to 7 weeks old) and 17 adult cats (1–2 years old) of either sex with no optical abnormalities were used in this study. This study was carried out in accordance with the recommendations of NIH guidelines, and all animal experimental protocols were approved by the Animal Care and Use Committee of Fudan University, which are similar to NIH guidelines, to minimize the usage of experimental animals, to relieve possible pain and to reduce unnecessary surgical procedures.

Four individual groups of cats were used, and they were normal kittens (*n* = 3), normal adult cats (*n* = 4), kittens deprived for 3 days (*n* = 16), adult cats deprived for 7 days (*n* = 13). They were all reared in the same 12 square meters space under 12-h light/dark cycle (deprived animals were eyelid sutured for 3 or 7 days; normal kittens and adult cats were reared for 3 and 7 days, respectively. Then, acute experiments of intrinsic signal optical imaging and/or single unit recording were performed in these groups of animals. Since the major technical challenge in this project came from the weak optical signals monocularly evoked in area 21a. To maximize the success rate, we performed the experiment in the following procedure. For each monocularly deprived animal, we first did optical imaging in area 21a. Only when orientation differential maps from the non-deprived eye were clear, so that OD evaluation based on two monocular signals was reliable, did we complete optical imaging in area 17. Only when the same evaluation in area 17 was reliable, did we count the data. If anyone of optical signals in the above areas was not good enough to enable same-animal comparison of these two areas, and we could not include the imaging data, we would move on to the electrophysiological recordings (or in some cases, after successful optical imaging). For example, in kittens, the success rate of optical imaging in this monocular deprivation project is 5 out of 16, and 13 of them provided single unit data.

Animals were initially anesthetized with ketamine (20 mg/kg) and sustained by 2.0∼3.0% isoflurane during the surgery. Atropine sulfate (0.04 mg/kg), cephalosporin (50 mg) and dexamethasone (2 mg/kg) were administered subcutaneously to reduce secretions, infection and edema, respectively. All incised tissues were permeated with lidocaine (2%). A venous cannulation was performed, and a mixture of gallamine triethiodide (10 mg/kg/h) and glucose (5%) in saline was infused intravenously to maintain paralysis. A tracheotomy was performed; the cat was artificially respired by a pulmonary pump (6025, UGO Basile, Italy) via a tracheal cannula, and end-tidal CO2 was monitored and kept at approximately 3.5∼4.0%. Anesthesia was maintained by 1.0∼2.0% isoflurane during optical imaging and electrophysiology. The heart rate was monitored and kept between 180 and 220 beats per minute. The body temperature was maintained at 38.5°C by a feedback-controlled heating pad (BME-461A, Institute of Biomedical Engineering, China).

The pupils of the cat were dilated with 1% atropine, and nictitating membranes were retracted with 5% neosynephrine. The eyes were refracted carefully and corrected with appropriate contact lenses. To reduce the amount of spherical aberration, artificial pupils (3 mm in diameter) were placed in the front of each eye.

Cat was then placed in a stereotaxic apparatus (Jiangwan II type, The Second Military Medical University, China). The visual cortex was exposed through craniotomy and durotomy at Horsley-Clarke coordinates L0–8, P0–10 for area 17 and L7–12, P1–7 for area 21a as described in our previous reports (Huang et al., 2006; Yu et al., 2008; Li et al., 2017). Area 21a was localized according to its relationship to the middle suprasylvian gyrus and lateral sulcus.

### Monocular Deprivation

Sixteen kittens and 13 adult cats were monocularly deprived. Cats were initially anesthetized with 2.5% isoflurane. After disinfecting the skin around the eye to be deprived, the eyelids were trimmed and sutured together with mattress stitches. Chloramphenicol ophthalmic ointment was administered to prevent infection. Monocularly deprived cats were raised identically to normal cats under a 12-h light/dark cycle, and the monocularly deprived and normal cats could eat, drink, run and play freely in a 12 square meter space with plenty of toys to enable sufficient visual stimulation. MD lasted for 3 days during the CP and 7 days in adult cats. At the end of the deprivation period, cats were anesthetized, the stitches were removed, and the lid margins were separated. Eyes were flushed with sterile saline and checked for clarity. Acute recording experiments were performed right after these.

### Intrinsic Signal Optical Imaging

Optical imaging was performed on area 21a and area 17 contralateral to the deprived eye. After exposure of the visual cortical area, a piece of artificial dura (0.005-inch Silicon Sheeting, Specialty Manufacturing, Inc., United States) was inserted between the cortex surface and the dura. It was then covered with 3% agarose to prevent drying, reduce pulsations and provide mechanical support. This acute window was sealed by a transparent cover glass and cemented to the skull by super dental bond (Super-Bond C&B, Sun Medical, Co., Japan). Optical signals were acquired from areas 21a and 17 to obtain a high-quality map.

Images of cortical reflectance were obtained using a digital CCD camera (1600 × 1200 pixels, 6.7 μm × 6.7 μm/pixel, 14-bit; PCO 1600, PCO AG, Germany). A Tandem-lens configuration (Nikkon 50 mm f/1.2, Nikon, Japan) was used to achieve maximal light yield and a shallow depth of focus.

A cortical vascular map was obtained under illumination of green light (546 nm). The camera was then focused 500 μm below the meninges, and functional orientation maps were then captured with red light (630 nm). Data acquisition was synchronous with the stimulus, which started 3 s before stimulus presentation, and a total of 10 frames at a frame rate of 1 Hz were recorded.

### *In vivo* Electrophysiology

Extracellular electric signals were recorded by a glass-coated tungsten microelectrode (3–5 MΩ). The microelectrode was positioned perpendicularly to the surface of the exposed cortex covered with agar and advanced through the cortex using a pulse motor Microdrive (SMX micromanipulator, Sensapex, Finland). Cells were recorded at cortical depths between 500 and 1500 μm. At the end of the experiment, the electrode was left in the targeted area 21a, and the animal was perfused and Nissl stained to reconstruct the electrode track to confirm its location in area 21a.

The signal was amplified (2400A, Dagan Corporation, United States), bandpass filtered (300 Hz–3 kHz for single-unit signals) and sampled at 10 kHz using a data acquisition system (CED Micro 1401, Cambridge Electronic Design, Ltd., United Kingdom) under the control of spike2 software (version 6; Cambridge Electronic Design, Cambridge, United Kingdom). Spikes were sorted by spike2 software and analyzed in MATLAB (MathWorks, Natick, MA, United States).

### Visual Stimuli

Stimuli were created using MATLAB with the Psychophysics Toolbox (Brainard, 1997; Pelli, 1997) and presented on a CRT monitor (FlexScan F931, Eizo Nanao Corporation, Japan; refresh rate 120 Hz, mean luminance ∼15 cd/m^2) placed 57 cm away from the cat’s eyes. One eye was covered by a custom-made shutter that could slide between contralateral and ipsilateral eyes. The cats in the experiment were stimulated monocularly by full field (30°^∗^40°) drifting sinusoidal gratings. Gratings were presented in 8 directions (for optical imaging) or 24 directions (for single-unit recordings), which were distributed uniformly from 0 to 360° (contrast 0.9; temporal frequency 2 Hz; spatial frequency 0.2–0.5 cycles/degree). The moving directions of the gratings were always orthogonal to the orientation.

Each trial of optical imaging consisted of a 3-s blank gray screen at the mean luminance of the gratings, a 4-s visual stimulus and a 16-s blank screen for recovery (23 s in total). Each trial of single-unit recordings consisted of a 1-s visual stimulus and a subsequent 1-s blank screen (2 s in total). Each identical stimulus was repeatedly shown 16 or 32 times in a session to increase the signal/noise ratio. The stimulus array in each trial was presented in a pseudo-random order to avoid any adaptation to the order of stimulus presentation.

### Data Analysis

Data were analyzed by custom-made scripts written in MATLAB. For single-unit signals, the firing rate of a neuron was averaged from 50 to 400 ms after the onset of the preferred orientation, and spontaneous responses were subtracted from the raw data. The preferred orientation was calculated based on a vector summation method:

$$\text{S}=\frac{\sum_{\text{k}}{\text{r}}_{\text{k}}\,{\text{e}}^{\text{i}\,2\,\theta_{\text{k}}}}{\sum_{\text{k}}{\text{r}}_{\text{k}}}$$

where θ_k_ is the direction of the drifting grating and r_k_ is the firing rate in that direction. Briefly, the responses of each cell to the different directions of the stimulus could be formulated as a series of vectors. The vectors were summed and normalized, and the angle of the resultant vector gives the preferred orientation.

For intrinsic signals, first frame analysis was applied to remove slow wave noise: all 10 frames in each trial were first subtracted and then divided by the mean of the first three frames (Rb, which is the reflectance of the cortex obtained when no stimulus was presented). The resultant frames (dR/R = R/Rb − 1) were defined as the orientation single-condition maps.

In most cases, we subtracted two single-condition maps elicited by orthogonal gratings (for example, 0 vs. 90°, 45 vs. 135°) to enhance the representation of the orientation-specific difference of the elicited response. Since the strongest intrinsic signal appeared 3∼4 s after the onset of the visual stimuli, we applied the subtraction on the 7th frame of the single-condition maps to generate a 0–90° (or 45–135°) differential map.

The global signal is a visually evoked cortical response. To calculate the strength of the global signal, the exposed visual cortical region was outlined as a region of interest (ROI). The global signal was the mean of all pixels in the ROI of the single-condition map.

**FIGURE 1:**
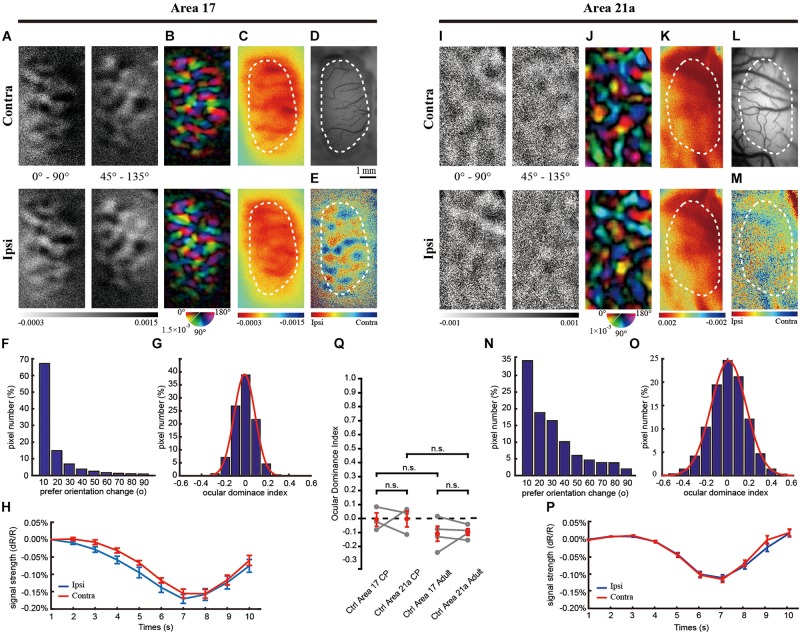
The same-animal comparison between ocular dominance (OD) maps in areas 17 **(A**–**H)** and 21a **(I**–**P)** in normal cats (**A**–**P** are from one typical cat, and **Q** illustrates data pairs of area 17 and area 21 from multiple animals). **(A,I)** Each row shows orientation differential maps evoked by monocular stimuli. Top: contralateral eye (contra). Bottom: ipsilateral eye (ipsi). Each column shows orientation differential maps with varying- orientation stimuli. Left: 0–90°. Right: 45–135°. **(B,J)** Orientation tuning is visualized by orientation polar maps, in which different colors represent the preferred stimulus orientation, and the gray scale represents the tuning strength of each pixel. Top: contralateral eye. Bottom: ipsilateral eye. **(C,K)** Global signal maps showing the averaged cortical response to stimuli with all orientations. Top: contralateral eye. Bottom: ipsilateral eye. **(D,L)** Blood vessel map. Scale bars: 1 mm. **(E,M)** OD maps of area 17 and area 21a. Red regions of the OD map represent domains dominated by the ipsilateral eye, and blue regions represent domains dominated by the contralateral eye. **(F,N)** The distribution of pixelwise differences of preferred orientation between the two eyes. **(G,O)** The distribution of the ocular dominance index (ODI) of pixels in the dashed regions in **(E,M)**, respectively. **(H,P)** The time course of the global signals in area 17 and area 21a for the two eyes. Each global signal represents the mean of all the pixels in the region of interest (ROI) (shown as dotted region in **C** or **K**). **(Q)** Statistical analysis of ODI in control animals. The mean ODI of all the pixels in the ROI was calculated as the ODI of this cortical area and shown as one individual point. *N* = 3 kittens during the critical period (CP) and *n* = 4 adult animals. Note that both area 17 and area 21a were imaged from the same animal, and the data points from the same animal are connected with lines. Data are the means ± SEM; paired-sample and two-sample *t*-tests: n.s., not significant.

**FIGURE 2:**
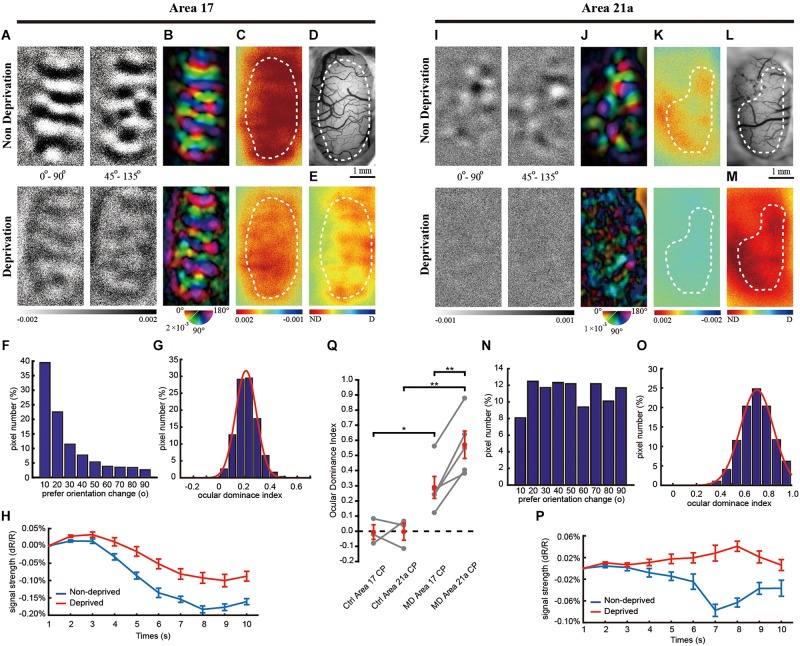
The same-animal comparison between OD maps in areas 17 **(A**–**H)** and 21a **(I**–**P)** in kittens monocularly deprived for 3 days (**A**–**P** are from one typical cat, and **Q** illustrates data pairs of area 17 and area 21 from multiple animals). **(A,I)** Each row shows orientation differential maps evoked by monocular stimuli. Top: non-deprived eye. Bottom: deprived eye. Each column shows orientation differential maps with varying-orientation stimuli. Left: 0–90°. Right: 45–135°. **(B,J)** Orientation tuning is visualized by orientation polar maps, in which different colors represent the preferred stimulus orientation, and the gray scale represents the tuning strength of each pixel. Top: non-deprived eye. Bottom: deprived eye. **(C,K)** Global signal maps showing the averaged cortical response to stimuli with all orientations. Top: non-deprived eye. Bottom: deprived eye. **(D,L)** Blood vessel map. Scale bars: 1 mm. **(E,M)** OD maps of area 17 and area 21a. Red regions of the OD map represent domains dominated by non-deprived eyes, and blue regions represent domains dominated by deprived eyes. **(F,N)** The distribution of pixelwise differences of preferred orientation between the two eyes. **(G,O)** The distribution of the ODI of pixels in the dashed regions in **(E,M)**, respectively. **(H,P)** The time course of the global signals in area 17 and area 21a for the two eyes. Each global signal represents the mean of all the pixels in the ROI (shown as dotted region in **C** or **K**). **(Q)** Statistical analysis of the OD shift in kittens. The mean ODI of all the pixels in the ROI was calculated as the ODI of this cortical area and shown as one individual point. *N* = 3 kittens for the control group and *n* = 5 kittens for the MD group. Note that area 17 and area 21a were imaged from the same animal, and the data points from the same animal are connected with lines. Data are means ± SEM; paired-sample and two-sample *t*-tests: ^∗^*p* < 0.05, ^∗∗^*p* < 0.01.

**FIGURE 3:**
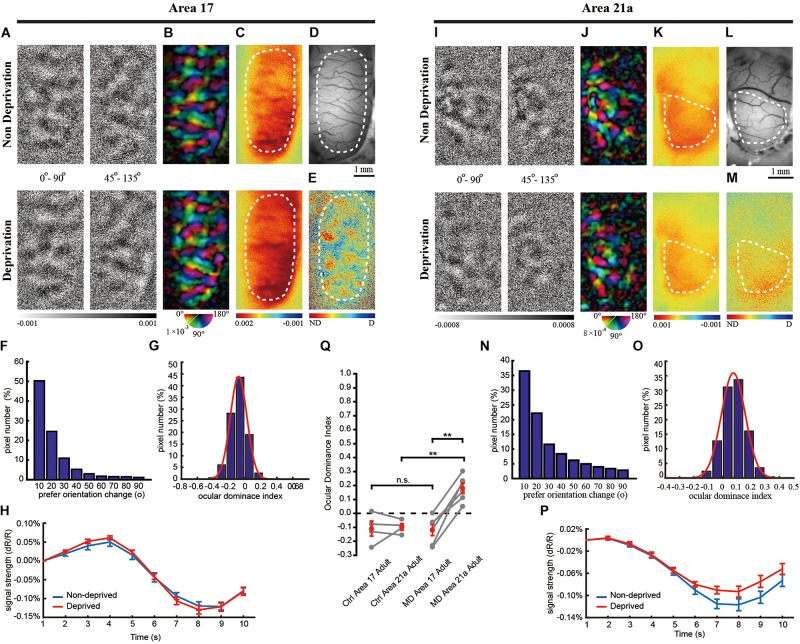
The same-animal comparison between OD maps in areas 17 **(A**–**H)** and 21a **(I**–**P)** in adult cats monocularly deprived for 7 days (**A**–**P** are from one typical cat, and **Q** illustrates data pairs of area 17 and area 21 from multiple animals). **(A,I)** Each row shows orientation differential maps evoked by monocular stimuli. Top: non-deprived eye. Bottom: deprived eye. Each column shows orientation differential maps with varying-orientation stimuli. Left: 0–90°. Right: 45–135°. **(B,J)** Orientation tuning is visualized by orientation polar maps, in which different colors represent the preferred stimulus orientation, and the gray scale represents the tuning strength of each pixel. Top: non-deprived eye. Bottom: deprived eye. **(C,K)** Global signal maps showing the averaged cortical response to stimuli with all orientations. Top: non-deprived eye. Bottom: deprived eye. **(D,L)** Blood vessel map. Scale bars: 1 mm. **(E,M)** OD maps of area 17 and area 21a. Red regions of the OD map represent domains dominated by non-deprived eyes, and blue regions represent domains dominated by deprived eyes. **(F,N)** The distribution of pixelwise differences of preferred orientation between the two eyes. **(G,O)** The distribution of the ODI of pixels in the dashed regions in **(E,M)**, respectively. **(H,P)** The time course of the global signals in area 17 and area 21a for the two eyes. Each global signal represents the mean of all the pixels in the ROI (shown as dotted region in **C** or **K**). **(Q)** Statistical analysis of the OD shift in the adult cat. The mean ODI of all the pixels in the ROI was calculated as the ODI of this cortical area and shown as one individual point. *N* = 4 animals for the control group and *n* = 6 animals for the MD group. Area 17 and area 21a were imaged from the same animal, and the data points from the same animal are connected with lines. Data are means ± SEM; paired-sample and two-sample *t*-tests: ^∗^*p* < 0.05, ^∗∗^*p* < 0.01.

The “cocktail blank” was defined as the averaged map of the single-condition maps elicited by all different orientation stimuli. All single-condition maps were divided by cocktail blank to emphasize the orientation specific signals. Vector summation of these revised single-condition maps, similar to that used for the single-unit data, was performed pixel by pixel to obtain the orientation polar maps. The OD map could be obtained by subtracting the global signal for the non-deprived (or ipsilateral) eye from that of the deprived (or contralateral) eye. An ocular dominance index (ODI) was calculated as (R_*non*-*depri*_−R_depri_)/(R_non–depri_ + R_depri_). The ODI ranges from −1 to 1, where a positive value indicates a non-deprived–eye bias and a negative value indicates a deprived-eye bias. The ODI was calculated pixel by pixel. In some pixels, the values of monocular eye response might be weaker than 0, and their response values would be set as 0, thus the ODIs of these pixels were the extreme value of −1 or 1. The ODI distribution of all the pixels in the ROI was illustrated in G&O of Figures 1–3 and the mean ODI of one region was shown in Q of Figures 1–3.

To quantify the similarity between two maps, two-dimensional cross-correlation coefficients (CCCs) were calculated. The CCC ranges from −1.0 to 1.0. Two spatially identical maps have a CCC of 1.0, and two complementary maps have a CCC of −1.0. If two maps do not have any correlation, their CCC is zero.

Paired and unpaired Student’s *t*-tests were used to determine the statistical significance. *p* < 0.05 was considered statistically significant. Data are presented as the mean ± standard error of the mean (SEM).

## Results

### OD in Area 17 and 21a in Control Cat

We first examined the similarity of response properties, such as orientation polar (OP) maps and ODI, between contralateral and ipsilateral eyes in area 17 and 21a in normal cats. This enabled us to determine the extent of the change in the maps after MD. As shown in Figures 1A,B, a typical case from a kitten exhibited identical orientation differential maps and orientation polar maps in area 17 produced by independent stimulation of the two eyes. The corresponding iso-orientation domain and pinwheel centers were at the same locations as those of the two orientation polar maps. To quantify the similarity of the preferred orientation for the left and right eye, we calculated the shift of the preferred orientation of each pixel between the preferred angles of the two orientation maps. More than 95% of the pixels in Figure 1B shift their preferred orientation by less than 20°, suggesting little change in the preferred orientation in the overall population. The OD map was tessellated with stripe-like territories (Figure 1E), and the ODI distribution of the pixels in this region is shown in Figure 1G. To assess the visually evoked cortical response of one specific eye without orientation preference, the global signal was routinely employed (see methods), which, in brief, was the cocktail of all orientation single-condition maps (also see similar analysis in Bonhoeffer et al., 1995 for cat, Antonella et al., 1999 for mouse, Ts’O et al., 1990 for monkey, Issa et al., 1999 for ferret). Due to the increased oxygen consumption, the responsive areas became darker after the onset of the stimuli, and the peak appeared at the 7th second. The global signals of the contralateral and ipsilateral eyes were nearly identical (contralateral: 0.13 ± 0.013% vs. ipsilateral: 0.14 ± 0.008%, *n* = 8 trials, *p* = 0.8193, *p* > 0.05, two-sample *t*-test, Figure 1H).

Overall, the statistics from all the animals recorded in area 17 showed the same results. The CCC between two preferred orientation angle maps was 0.76 ± 0.06 (*n* = 3) in kittens and 0.84 ± 0.03 (*n* = 4) in adult cats. The mean ODI was −0.0065 ± 0.0480 in kittens (*n* = 3, Figure 1Q) and −0.1085 ± 0.0542 in adult cats (*n* = 4, Figure 1Q).

Similarly, orientation differential maps and orientation polar maps in area 21a from the two eyes shared similar layouts. The CCC between the preferred orientation angle maps for the left and right eye was 0.45 ± 0.04 (*n* = 3) in kittens and 0.43 ± 0.10 (*n* = 4) in adult cats.

The OD organization in area 21a was distinctly different from that of area 17. Although previous electrophysiological studies have suggested that many individual cells in area 21a are dominated by inputs from one eye (Dreher et al., 1993, 1996), the macroscopic organization of OD in area 21a was absent in our images, with no stripe-like territories (Figure 1M). Overall, the mean ODI was −0.0037 ± 0.0561 in kittens (*n* = 3, Figure 1Q) and −0.0963 ± 0.0234 in adult cats (*n* = 4, Figure 1Q). No significant difference in ODI was found between areas 17 and 21a in the control group (*p* = 0.2946, *p* > 0.05 for kittens, *n* = 3; and *p* = 0.3344, *p* > 0.05 for adult cats, *n* = 4; paired-sample *t*-test, Figure 1Q).

### OD Plasticity in Areas 17 and 21a in the Critical Period

In area 17, MD for 3 days in the CP resulted in an apparent decrease in the response of the deprived eye. Although the orientation differential maps (Figure 2A) and polar map (Figure 2B) of the deprived eye were less distinct than those of the non-deprived eye, the patterns of the orientation polar maps of the two eyes were very similar (also see Godecke and Bonhoeffer, 1996; Crair et al., 1998). Sixty percent of the pixels shifted their preferred orientation by less than 20° (Figure 2F). The above findings demonstrate that MD did not change the overall pattern of the orientation map in area 17. The global responses of the non-deprived eye were much stronger than those of deprived eye (non-deprived eye: 0.19 ± 0.009% vs. deprived eye: 0.09 ± 0.007%, *n* = 8 trials, *p* = 0.00048, *p* < 0.01, two-sample *t*-test, Figure 2H). These results were consistent with those of earlier studies (Crair et al., 1997; Jaffer et al., 2012).

A rapid and sharp decrease in the deprived-eye response was observed in area 21a. The response of the deprived eye was not measurable in the orientation differential maps, and thus, the orientation polar map was deteriorated (Figures 2I,J). The distribution of the shifts of the preferred orientation was relatively flat, and 80% of the pixels shifted their preferred orientation by more than 20°. The overall global signal of the deprived eye showed almost no response after the onset of visual stimulation (non-deprived eye: 0.08 ± 0.01% vs. deprived eye: −0.03 ± 0.009%, *n* = 8 trials, *p* = 0.00034, *p* < 0.01, two-sample *t-*test, Figure 2P), and the ODI distribution of all the pixels in this region showed a great shift toward non-deprived eye preference (Figure 2O). Another imaging case from a monocularly deprived kitten is shown in Supplementary Figure S1, illustrating a much greater ODI shift in area 21a than the one in area 17.

For all the animals tested, the CCC between two preferred orientation angle maps is 0.78 ± 0.07 (*n* = 5) in area 17 and 0.13 ± 0.03 (*n* = 5) in area 21. Compared to the control group, brief MD in the CP caused a strong OD shift to the non-deprived eye in area 17 (ODI = 0.289 ± 0.073, *n* = 3 and 5, control and MD, respectively, *p* = 0.029, *p* < 0.05, two-sample *t*-test, Figure 2Q) and area 21a (ODI = 0.571 ± 0.090, *n* = 3 and 5, control and MD, respectively, *p* = 0.0053, *p* < 0.01, two-sample *t*-test, Figure 2Q). It is important that area 21a had a greater ODI shift than area 17 based on the same-animal comparison (*p* = 0.0044, *p* < 0.01, *n* = 5, paired-sample *t*-test).

**FIGURE 4:**
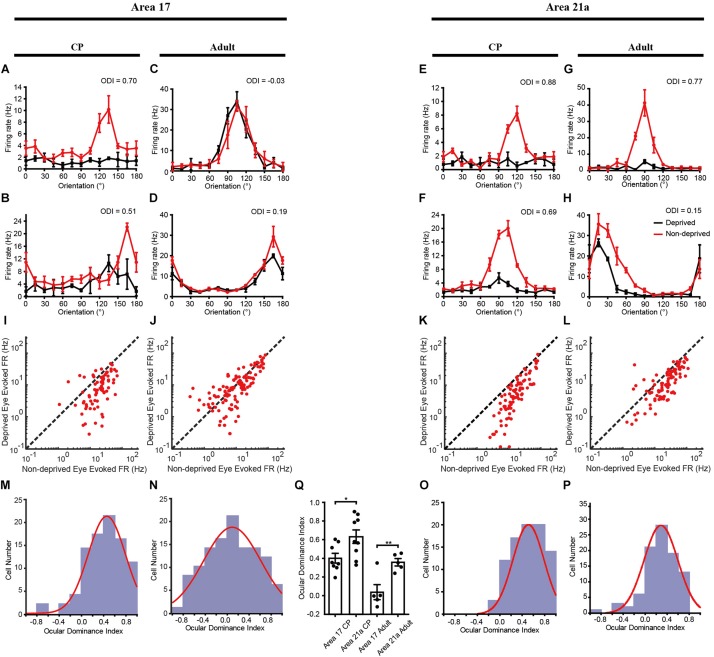
Electrophysiological evidence of MD effect on cats of different ages in areas 17 and 21a. Orientation-tuning curves of example neurons in area 17 **(A–D)** and area 21a **(E–H)** to stimuli presented to the deprived eye (black lines) and the non-deprived eye (red lines). A comparison of the firing rates between the deprived and non-deprived eyes in area 17 (**I**, during CP; **J**, in adulthood) and area 21a (**K**, during CP; **L**, in adulthood). Each red dot represents a single neuron. Ocular dominance index distribution of all the recorded cells in area 17 **(M,N)** and 21a **(O,P)** from cats monocularly deprived during the CP (3 days, **M,O**) or in the adult (7 days, **O,P**). The red curve indicates a Gaussian fit to the data. **(Q)** Statistical analysis of the single-unit recording results on the ODI shift across animals. Each point represents the mean ODI from the cortical area of one animal, and the mean and standard error of ODI across animals is shown as a bar plot. ^∗^*p* < 0.05, ^∗∗^*p* < 0.01.

In accordance with the optical imaging results, electrophysiological recordings also showed that the OD plasticity was stronger in area 21a. After 3 days of MD, recordings were made from 87 cells from 8 kittens in area 17 and 89 cells from 9 kittens in area 21a. Four representative cells are shown, and they unanimously demonstrated a strong preference for the non-deprived eye (Figures 4A,B,E,F). For all the recorded cells, a comparison of the firing rate between the deprived and non-deprived eye is shown in Figures 4I,K. In both area 17 (Figure 4I) and area 21a (Figure 4K), most of the neurons showed lower responsiveness to stimulation of the deprived eye (below the diagonal line), illustrating strong ODI shifts toward non-deprived eye dominance. Importantly, the overall shift of the ODIs in area 21a was significantly stronger than that in area 17 (area 17: 0.414 ± 0.036, *n* = 87 neuron vs. area 21a: 0.512 ± 0.029, *n* = 89 neurons *p* = 0.012, *p* < 0.05, two-sample *t*-test, Figures 4M,O). The mean ODI of each individual kitten is also illustrated in Table 1, and across animals, the mean shift of ODI in area 21a was also significantly stronger than that in area 17 (area 17: 0.403 ± 0.052, *n* = 8 kittens vs. area 21a: 0.638 ± 0.082, *n* = 9 kittens, *p* = 0.026, *p* < 0.05, two-sample *t*-test, Figure 4Q). The degree of this OD shift was comparable with the OD shift observed in optical imaging.

**TABLE 1:** Effects of MD measured by electrophysiology.

**Period**	**Cat No.**	**ODI Area17**	**ODI Area21a**
CP	CAT20141107	0.52 ± 0.064	0.80 ± 0.077
	CAT20141115	0.32 ± 0.070	0.58 ± 0.047
	CAT20141122	0.30 ± 0.058	0.37 ± 0.103
	CAT20150124	0.20 ± 0.156	0.34 ± 0.038
	CAT20141103	0.61 ± 0.046	
	CAT20141129	0.36 ± 0.135	
	CAT20150606	0.58 ± 0.058	
	CAT20140726	0.34 ± 0.060	
	CAT20140514		0.82 ± 0.049
	CAT20140529		0.89 ± 0.040
	CAT20140811		0.49 ± 0.053
	CAT20141018		0.91 ± 0.039
	CAT20150322		0.59 ± 0.046
Adult	CAT20141026	−0.04 ± 0.069	0.44 ± 0.057
	CAT20141227	0.002 ± 0.0727	0.35 ± 0.138
	CAT20150523	0.35 ± 0.084	
	CAT20150716	−0.002 ± 0.0761	
	CAT20150815	−0.12 ± 0.091	
	CAT20150103		0.46 ± 0.063
	CAT20150418		0.31 ± 0.070
	CAT20150425		0.24 ± 0.042

### MD Induced OD Shifts in Area 21a but Not in Area 17 in the Adult Cat

As described previously, susceptibility to MD disappeared beyond the CP of the cat (Hubel and Wiesel, 1970). In our experiment, we performed 7 days of MD on cats (1∼2 years old). In area 17, the magnitude of the response from the deprived eye in the orientation differential maps (Figure 3A) and polar map (Figure 3B) was similar to that from the non-deprived eye. More than 75% of the pixels in Figure 3B shifted their preferred orientation by less than 20° (Figure 3F). The global signals of the non-deprived eye and the deprived eye were nearly equal (non-deprived eye: 0.12 ± 0.010% vs. deprived eye: 0.13 ± 0.011%, *n* = 16 trials, *p* = 0.2398, *p* > 0.05, two-sample *t-*test, Figure 3H).

Interestingly, OD plasticity persisted in area 21a beyond the CP. MD led to a moderate decrease in the orientation differential maps (Figure 3I) and polar map (Figure 3J) of the deprived eye. Forty-five percent of the pixels in Figure 3J shifted their preferred orientation by more than 20° (Figure 3N). The global signals of the non-deprived eye were stronger than those of the deprived eye (non-deprived eye: 0.12 ± 0.008% vs. deprived eye: 0.09 ± 0.007%, *n* = 28 trials, *p* = 0.0243, *p* < 0.05, two-sample *t*-test, Figure 3P). Another imaging case from a monocularly deprived adult cat is shown in Supplementary Figure S2, illustrating a significant ODI shift in area 21a but not in area 17.

On average, the CCC between two preferred orientation angle maps is 0.70 ± 0.06 (*n* = 6) in area 17 and 0.36 ± 0.08 (*n* = 6) in area 21. MD in the adult cat caused a significant OD shift to the non-deprived eye in area 21a (Figure 3Q, ODI = 0.180 ± 0.037, *n* = 4 and 6 cats, control and MD, respectively; *p* = 0.00056, *p* < 0.01, two-sample *t*-test) but not in area 17 (ODI = −0.120 ± 0.040, *n* = 4 and 6 cats, control and MD, respectively; *p* = 0.8686, *p* > 0.05, two-sample *t-*test). Ultimately, the OD shift evoked by MD in area 21a was much greater than that in area 17 based on the same-animal comparison (*p* = 0.00070, *p* < 0.01, *n* = 6 cats, paired-sample *t*-test).

Single-unit recordings confirmed a significant OD shift in area 21a over the CP. Cats between 1 and 2 years old were monocularly deprived for 7 days, and then recordings were made from 114 cells acquired from 5 cats in area 17 and 93 cells acquired from 5 cats in area 21a. Four representative cells showed a weaker preference for the non-deprived eye (Figures 4C,D,G,H). In area 21a (Figure 4L), but not in area 17 (Figure 4J), most of the neurons showed lower responsiveness to stimulation of the deprived eye. The ODI distribution of cells from area 21a was clearly biased compared to that from 17 (area 17: 0.082 ± 0.043, *n* = 114 neurons vs. area 21a: 0.289 ± 0.032, *n* = 93 neurons, *p* = 0.00027, *p* < 0.01, two-sample *t*-test, Figures 4N,P). The mean ODI of each individual animal is also illustrated in Table 1, and across animals, the mean shift of ODI in adult area 21a was also significantly stronger than that in adult area 17 (area 17: 0.038 ± 0.081, *n* = 5 adult cats vs. area 21a: 0.360 ± 0.040, *n* = 5 adult cats, *p* = 0.0075, *p* < 0.01, two-sample *t*-test, Figure 4Q).

## Discussion

In this study, we examined the OD plasticity in areas 17 and 21a of cats within, as well as beyond, the classical CP by intrinsic signal optical imaging and single-unit recordings. We found that (1) slab-like OD maps could not be identified in area 21a; (2) in the classical CP, OD shifts evoked by 3 days’ MD were greater in area 21a than in area 17, suggesting a greater OD plasticity in area 21a; and (3) beyond the CP, 7 days’ MD could elicit significant MD shifts in area 21a but not in area 17, suggesting a prolonged plastic window for area 21a, although the shift in area 21a of the adult cat was much weaker than the one in the CP.

The major technical challenge of this study came from the weak optical signal in area 21a evoked by monocular visual stimulation. It has been shown that the orientation strength signal of area 21a is approximately 1/4 that of area 18 (Villeneuve et al., 2009), and the signal evoked monocularly would be even weaker. In fact, imaging and analysis based on weak signals are not reliable, and thus, the inter-individual variability of intrinsic-signal optical imaging is large. To overcome this problem, we set strict criteria based on two important controls. First, non-deprived eye-driven optical maps must demonstrate black and white mosaic-like orientation differential maps to validate an optically responsive area 21a. Based on this, deprived eye-driven responses could be compared to calculate the relative OD preference. Second, area 17 of the same cat must be imaged, clear orientation differential maps must be obtained, and then the ODI of area 17 would be compared with that in area 21a. This control is important to reduce inter-animal variability, and thus, in the imaging data, a paired *t* test was performed; since one of our major goals was to examine the difference in OD plasticity between area 17 and area 21a. The technical difficulties and strict criteria led to a low yield rate; approximately 1/3 of our monocularly deprived animals were able to provide reasonable imaging data, with the remaining animals used for electrophysiological recordings in area 17 and/or area 21a.

Functional organization has been extensively explored in the primary sensory cortices, while the OD map in area 21a of the cat has not been previously examined. In fact, the columnar structure of orientation preference has been well described by the intrinsic signal optical imaging technique, demonstrating smaller iso-orientation domains in area 21a compared to the one in area 17 (Huang et al., 2006; Villeneuve et al., 2009). Similar to orientation selectivity, the neurons in area 21a also have strong eye preferences, and statistically, a broad distribution from purely ipsilateral to contralateral eye preference was demonstrated by previous single-unit recordings (Dreher et al., 1996, also see our Figures 4O,P). However, no organized OD map could be obtained in our study, suggesting that neurons with similar OD preferences might not cluster in area 21a. In fact, in V4 of the macaque monkey, which is analogous to area 21a of the cat, no patch-like OD map has ever been reported, although orientation and some other feature maps are clearly depicted in this part of the visual cortex (Tanigawa et al., 2010; Li et al., 2013; Lu et al., 2018).

This study demonstrated, for the first time, OD plasticity in area 21a evoked by MD during and beyond the CP. Similar to the OD plasticity in area 17, OD plasticity in area 21 did decrease with age, with a greater OD shift following 3-day MD during the CP than that following 7-day MD in older cats, suggesting a general reduction of cortical plasticity, which may originate from multiple factors during development (Sur and Leamey, 2001).

Besides the similarity, the OD plasticity in area 21a is significantly different from the one in area 17: the OD shift was greater in area 21a during the CP, while beyond the CP, it is even more surprising that a change in the inputs to area 21a provoked plasticity in this area at ages when antecedent region of area 17 appeared unchanged following MD. What mechanisms in area 21a may be responsible for this enduring plasticity? One possibility might lie in the structural difference. For example, the CP of mice primary visual cortex is closely related to the maturation of inhibitory neurons, and transplantation of the precursors of inhibitory neurons into adult cortex could lead to the second CP (Southwell et al., 2010). A recent study also showed that excitatory activities propagated sequentially to downstream areas instructed stagewise circuit maturation in the entorhinal-hippocampal hierarchical neural circuits (Donato et al., 2017). Multiple other factors, such as the gradients in cytoarchitecture, gene expression, and long-range axonal connectivity, may also account for the unique timing and duration of the CPs of different cortical areas (Chomiak and Hu, 2017; Fulcher et al., 2019). It is possible that different cortical areas may have different properties of these factors, and thus behave differently in the CP. Another possibility might come from the strength of functional change, and actually, it was already shown in the CP that area 21a suffered more severe reduction of deprived eye evoked response. It is well known that to enable long term synaptic plasticity, post-synaptic membrane potential must depolarize beyond certain threshold to evoke either LTP or LTD. In adulthood, although the MD effect declined across age, the greater change of responsiveness in area 21a might still reach the threshold to evoke a long-lasting functional shift while the milder one in area 17 failed.

The following question is how can area 21a achieve enhanced functional shift? First, it might come from the intrinsic intra-cortical circuitry within area 21a. As we mentioned above, in area 21a, we could not illustrate stripe-like clustered structure of OD map. It indicates that neighboring neurons might have diverse ODIs, and thus, the local intra-cortical network receives the inputs of both eyes. This in turn suggests a stronger inter-ocular competition, and it facilitates a higher OD plasticity (Cynader, 1982; Trachtenberg, 2015). Similar results have shown that the orientation plasticity is higher near the orientation pinwheels (where neurons with diverse orientation preferences are located within 100 microns) but weaker at the centers of the iso-orientation domains in cat area 17 (Dragoi et al., 2001) and area 21a (Meng et al., 2018). Second, plasticity in area 21a may also be inherited from upstream regions. Changes in the connections between higher-order cortices and the ascending inputs from early visual areas are accumulated and amplified progressively in multiple stages of processing (Yang and Maunsell, 2004; Bi et al., 2011; Dhruv and Carandini, 2014). This idea is consistent with several studies in the hierarchical visual system. For OD plasticity, during the CP, the extragranular layers are much more pronouncedly affected than layer IV (bottom layer, which receives inputs from LGN and sends outputs to other layers) by short-term MD in area 17 of kittens (Trachtenberg et al., 2000). In animal models of amblyopia, several electrophysiological studies in the CP also show that the magnitude of the physiological anomalies in higher-order visual areas are more severe than that in lower-order visual areas (Kiorpes et al., 1998; Schroder et al., 2002; El-Shamayleh et al., 2010; Bi et al., 2011). Similar phenomena have also been described in other forms of functional plasticity, such as orientation adaptation, where enhanced effects of adaptation propagate across different visual cortices (Kohn and Movshon, 2004; Dhruv and Carandini, 2014; Li et al., 2017).

However, a simple bottom–up cascading effect of functional plasticity in the hierarchical system could not explain our results and some other findings. In the adult cat, there was no significant OD shift in area 17, but the upper-level area 21a showed a significant shift toward non-deprived eye dominance, illustrating an extended time window of plasticity. Thus, the OD shift in adult area 21 might not be directly inherited and cascaded from the one in area 17. Furthermore, previous studies in another extrastriate cortex, LS, suggested even more complicated results. MD evoked a similarly strong OD shift in LS compared to the one in area 17 during the CP, but the OD plasticity in this higher-order cortex ended much sooner in the later stage, illustrating a shortened time window of plasticity (Jones et al., 1984). Why do the higher-order visual cortices behave differently when receiving the same inputs from the bottom-level primary visual cortex?

We believe that the feature-specific information processing pathway may play a major role in this phenomenon. Different features emerge by following different neural networks. For example, the OD preference of the neurons in the primary visual cortex emerges from inputs of purely monocularly driven neurons in the LGN. Similarly, an emerged feature may also become strengthened or weakened downstream depending on the network wiring. In fact, the visual information in the primary visual cortex is transferred along the ventral and dorsal pathways, and the adaptation effect may differ between the two. For example, the orientation adaptation effect is not enhanced from area 17 to the PMLS area, but the direction adaptation effect is; from area 17 to 21a, the orientation adaptation effect is enhanced, but the direction adaptation effect remains the same (Li et al., 2017), suggesting a feature-specific transferring effect along different visual streams. In this study, we demonstrated that OD plasticity could be enhanced or extended from area 17 to area 21a (the gateway of the ventral pathway), while previous studies have shown weaker plasticity in the PMLS area than in area 21a in strabismic cats (Schroder et al., 2002) or a shortened time window of OD plasticity in LS of MD cats (Jones et al., 1984). To elucidate this possible mechanism, more detailed investigations are desirable in the future.

## Data Availability Statement

The datasets generated for this study are available on request to the corresponding author.

## Ethics Statement

This study was carried out in accordance with the recommendations of NIH guidelines, and all animal experimental protocols were approved by the Animal Care and Use Committee of Fudan University, which are similar to NIH guidelines, to minimize the usage of experimental animals, to relieve possible pain and to reduce unnecessary surgical procedures.

## Author Contributions

JW and HY conceived of the project. JW, AJ, and TY performed the optical imaging recordings. JW performed the single-unit recordings. JW, ZN, and AJ performed the data analysis. ZN and HY wrote the manuscript.

## Conflict of Interest

The authors declare that the research was conducted in the absence of any commercial or financial relationships that could be construed as a potential conflict of interest.

## Supplementary Material

FIGURE S1 A comparison of the effect of 3 days’ MD between area 17 **(A,B,E,F)** and area 21a **(C,D,G,H)** in the same kitten. **(A,C)** Each row shows orientation differential maps evoked by monocular stimuli. Top: non-deprived eye. Bottom: deprived eye. Each column shows orientation differential maps with varying-orientation stimuli. Left: 0–90°. Right: 45–135°. **(B,D)** Orientation tuning is visualized by orientation polar maps, in which different colors represent the preferred stimulus orientation, and the gray scale represents the tuning strength of each pixel. Top: non-deprived eye. Bottom: deprived eye. **(E,G)** The distribution of pixelwise differences of preferred orientation between the two eyes. **(F,H)** The distribution of the ODI of pixels in the dashed regions in (e,m), respectively.

FIGURE S2 A comparison of the effect of 7 days’ MD between area 17 **(A,B,E,F)** and area 21a **(C,D,G,H)** in the same adult animal. **(A,C)** Each row shows orientation differential maps evoked by monocular stimuli. Top: non-deprived eye. Bottom: deprived eye. Each column shows orientation differential maps with varying-orientation stimuli. Left: 0–90°. Right: 45–135°. **(B,D)** Orientation tuning is visualized by orientation polar maps, in which different colors represent the preferred stimulus orientation, and the gray scale represents the tuning strength of each pixel. Top: non-deprived eye. Bottom: deprived eye. **(E,G)** The distribution of pixelwise differences of preferred orientation between the two eyes. **(F,H)** The distribution of the ODI of pixels in the dashed regions in (e,m), respectively.
