# Supplementary figures and images for: Ocular Dominance Plasticity of Areas 17 and 21a in the Cat
Source: Front Neurosci. 2019 Oct 11;13:1039. doi: 10.3389/fnins.2019.01039 (PMC6797596; doi:10.3389/fnins.2019.01039)

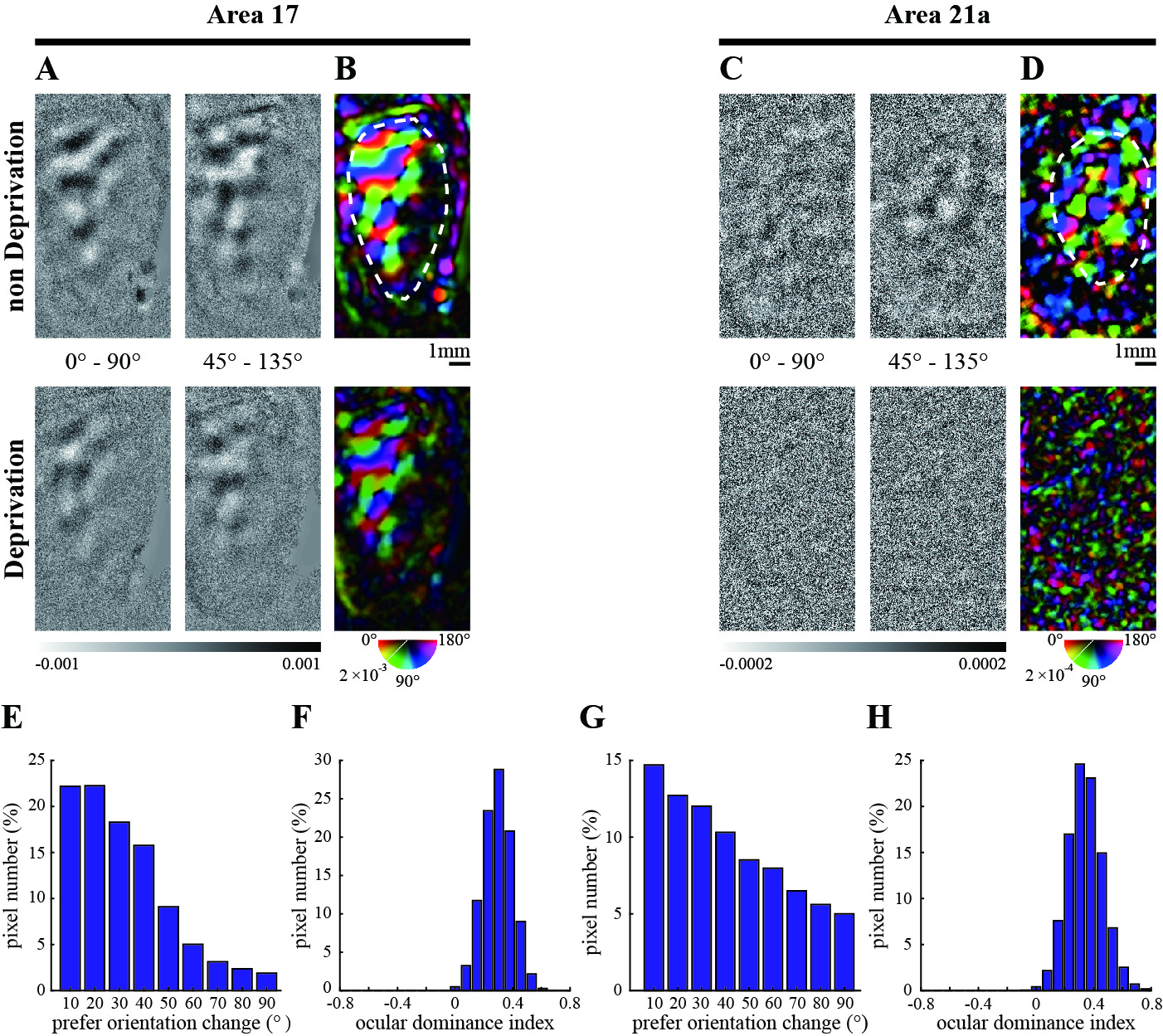

Supplement: FIGURE S1 — A comparison of the effect of 3 days’ MD between area 17 (A,B,E,F) and area 21a (C,D,G,H) in the same kitten. (A,C) Each row shows orientation differential maps evoked by monocular stimuli. Top: non-deprived eye. Bottom: deprived eye. Each column shows orientation differential maps with varying-orientation stimuli. Left: 0–90°. Right: 45–135°. (B,D) Orientation tuning is visualized by orientation polar maps, in which different colors represent the preferred stimulus orientation, and the gray scale represents the tuning strength of each pixel. Top: non-deprived eye. Bottom: deprived eye. (E,G) The distribution of pixelwise differences of preferred orientation between the two eyes. (F,H) The distribution of the ODI of pixels in the dashed regions in (e,m), respectively. [file Image_1.JPEG]

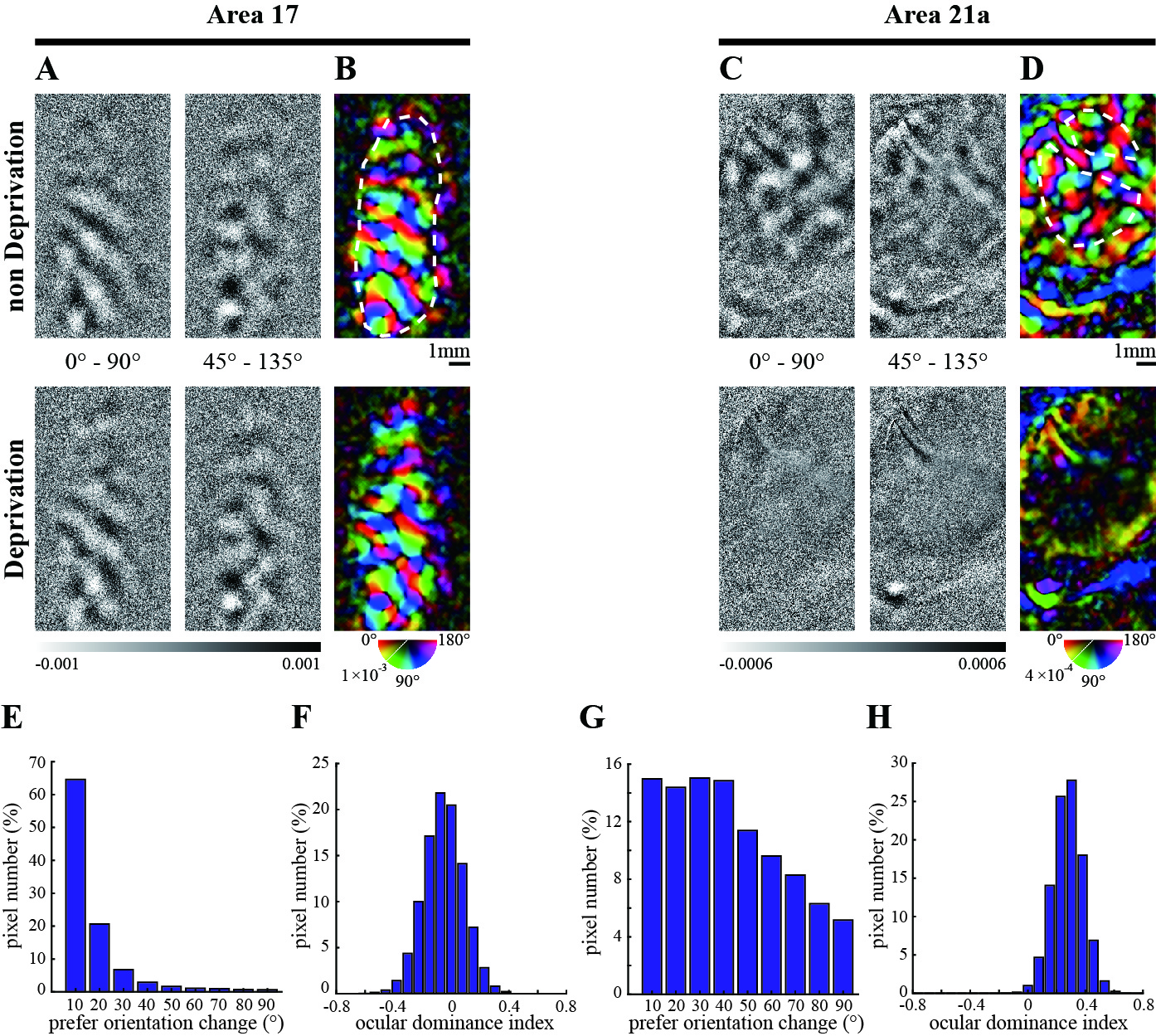

Supplement: FIGURE S2 — A comparison of the effect of 7 days’ MD between area 17 (A,B,E,F) and area 21a (C,D,G,H) in the same adult animal. (A,C) Each row shows orientation differential maps evoked by monocular stimuli. Top: non-deprived eye. Bottom: deprived eye. Each column shows orientation differential maps with varying-orientation stimuli. Left: 0–90°. Right: 45–135°. (B,D) Orientation tuning is visualized by orientation polar maps, in which different colors represent the preferred stimulus orientation, and the gray scale represents the tuning strength of each pixel. Top: non-deprived eye. Bottom: deprived eye. (E,G) The distribution of pixelwise differences of preferred orientation between the two eyes. (F,H) The distribution of the ODI of pixels in the dashed regions in (e,m), respectively. [file Image_2.JPEG]
